# Determination of Reference Intervals for Serum Total Calcium in the Vitamin D-Replete Pediatric Population

**DOI:** 10.1210/jc.2013-3105

**Published:** 2013-11-11

**Authors:** Jeffrey D. Roizen, Vipul Shah, Michael A. Levine, Dean C. Carlow

**Affiliations:** Division of Endocrinology and Diabetes (J.D.R., M.A.L.) and Division of Pathology and Laboratory Medicine (V.S., D.C.C.), The Children's Hospital of Philadelphia, and University of Pennsylvania Perelman School of Medicine, Philadelphia, Pennsylvania 19104

## Abstract

**Context:**

Widespread vitamin D insufficiency raises concerns regarding the reliability of reference intervals for serum calcium.

**Objective:**

We sought to determine the reference intervals for serum total calcium in pediatric subjects without vitamin D [25-hydroxyvitamin D [25(OH)D]] deficiency [20 ng/mL (50 nmol/L)].

**Design and Participants:**

This was a retrospective study of laboratory data obtained from all patients at The Children's Hospital of Philadelphia from July 1, 2011, through June 30, 2012. Patients in the renal unit, the endocrine unit, or a critical care unit were excluded. Total serum calcium was determined using a colorimetric assay and serum 25(OH)D was determined by liquid chromatography tandem mass spectrometry. We ascertained 4629 subjects who had a serum 25(OH)D between 20 and 80 ng/mL (50–200 nmol/L) and a serum calcium level determined within 30 days of the 25(OH)D measurement. For comparison, we used data from an unselected cohort of patients (n = 106 220).

**Results:**

Parametric analyses generated age-specific reference intervals for serum total calcium for each of several age groups (0–90 d old, 91–180 d old, 181–365 d old, 1–3 y old, 4–11 y old, and 12–19 y old). A two-way ANOVA with Tukey's correction showed significant differences between the lower limits of normal (*P* < .001) and the normal range (*P* < .001) but not for the upper limit of normal for these subjects compared with unselected subjects. Student's *t* tests revealed significant differences at all ages between calcium concentrations in those with 25(OH)D values between 20 and 30 ng/mL and those with 25(OH)D values between 30 and 80 ng/mL.

**Conclusions:**

These reference intervals refine previous normal ranges that likely included subjects with vitamin D deficiency.

The results of biochemical assays provide critical, often determinative components of medical decision making. Hence, normal reference intervals are critical to accurate interpretation of patient values. Reference intervals are conventionally determined using data sets comprised of test results obtained for a specific apparently healthy population and generating 95% confidence interval to define the normal range (1). The assumption underlying this approach is that only a small proportion of a normal population will consist of subjects with an abnormal test result, and thus, the effect of these outliers will not influence the final reference interval. This assumption has recently been challenged by the recognition that reference intervals for TSH were skewed because of the inclusion of subjects with biochemical hypothyroidism and mildly elevated TSH levels (2–4). These insights led to important revisions in the normal reference intervals for TSH in the adult population.

Because vitamin D [25-hydroxyvitamin D (25[OH]D)] deficiency (25[OH]D < 20 ng/dL (50 nmol/L)] and insufficiency [25(OH)D 30 ng/dL (75 nmol/L)] can reduce calcium absorption and cause hypocalcemia, we hypothesized that the high prevalence of vitamin D deficiency in the pediatric population (5) might affect serum calcium reference intervals. Here we report an innovative approach to determination of age-adjusted reference intervals for serum calcium excluding subjects with vitamin D deficiency. Our results provide refined reference intervals for calcium and show that many children and adolescents with serum concentrations of 25(OH)D between 20 and 30 ng/dL (50–75 nmol/L) have mildly depressed serum total calcium concentrations that skew calculated reference intervals.

## Materials and Methods

We measured total serum calcium by a colorimetric assay with the Ortho VITROS 5, 1 FS automated chemistry system (Ortho Clinical Diagnostics). This assay uses calibrators traceable to the certified National Institute of Standards and Technology reference material. The reportable range is from 1.0 to 14.0 mg/dL (0.3–3.5 mmol/L). The between-day coefficient of variation (22 d) is 1.4% and 1.6% at concentrations of 8.9 and 12.6 mg/dL (2.2–3.2 mmol/L), respectively. HPLC coupled with tandem mass spectrometry was used to measure serum total 25(OH)D based on the procedure of Maunsell et al (7) (2005) with modifications (6). The assay gave a linear response from 1.3 to 135 ng/mL (3.2–337 nmol/L) for both 25-hydroxyvitamin D_2_ [25(OH)D_2_] and 25-hydroxyvitamin D_3_[25(OH)D_3_]. The limit of quantitation was 1.3 ng/mL (3.2 nmol/L) for both compounds. The interassay variation was measured for both compounds by measuring the metabolite concentrations of three spiked serum specimens on each of 38 different days. The coefficients of variation for 25(OH)D_2_ were 10.0%, 9.0%, and 7.3% at 18, 35, and 100 ng/mL (45, 87, and 250 nmol/L), respectively, and for 25(OH)D_3_, 4.2%, 4.9%, and 4.8% at 21, 43, and 60 ng/mL (52, 107, and 149.8 nmol/L), respectively (7). Certified reference material and external quality control samples were analyzed to meet the standards outlined by the National Institute of Standards and Technology. Validation steps included recovery and both precision and accuracy under inter- and intraday variation limit of detection and analysis of each analyte over a linear range as described in Clinical and Laboratory Standards Institute guidelines. This assay detects both 25(OH)D_2_ and 25(OH)D_3_, and serum concentrations of 25(OH)D refer to the total concentrations of both metabolites.

We reviewed serum concentrations of total calcium and 25(OH)D that had been determined during the calendar year July 1, 2011, through June 30, 2012, from all patients at The Children's Hospital of Philadelphia (CHOP, n = 6868 unique patients). This population was drawn from all visits to CHOP during this time period, consisting of approximately 28 000 inpatient admissions and 1 140 000 outpatient visits. We included patients who had a serum 25(OH)D concentration between 20 ng/mL and 80 ng/mL measured within 30 days of their serum calcium measurement (n = 5449). We excluded patients admitted to the renal unit, the endocrine unit, or any critical care unit, cardiac intensive care unit (ICU), pediatric intensive care unit, and neonatal intensive care unit. These criteria yielded 4629 unique calcium values for 4629 patients. For comparison, values for all unique calcium measurements without any selection of patients were generated simultaneously (n = 106 220 unique values). We used EPEvaluator version 9 (Data Innovations, Inc) in accordance with Clinical and Laboratory Standards Institute guidelines to generate age-specific normal reference intervals. The CHOP Institutional Review Board determined that this study was exempt from institutional review board approval because it included only deidentified data.

## Results

**Figure 1.**
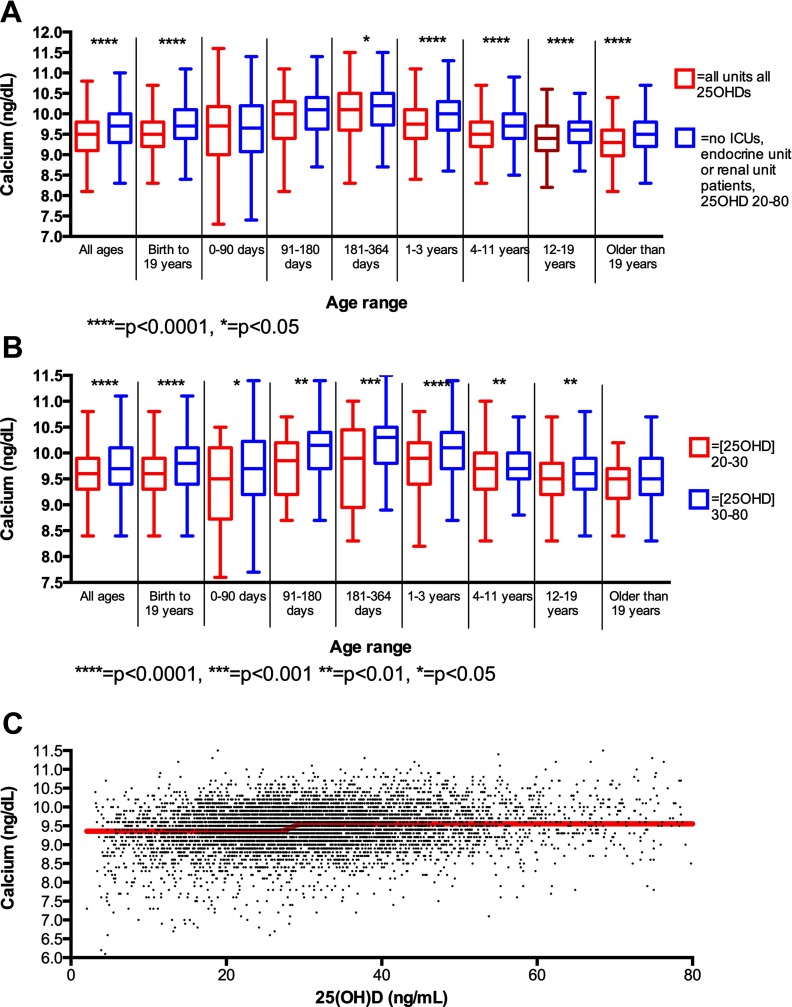
Calcium concentrations by age and 25(OH)D. A, Calcium concentrations (nanograms per deciliter) in unselected subjects compared with subjects not on the endocrine unit, not on the renal unit, and not in an ICU with 25(OH)D between 20 and 80 ng/mL. B, Calcium concentrations (nanograms per deciliter) eliminating patients with endocrine or renal diagnoses, and in ICUs stratified by 25(OH)D. C, Unselected subjects with fitted dose-response curve; this curve revealed an inflection point at 28.4 with 95% confidence intervals ranging from 27.7 to 29.0, and an R^2^ value of 0.022.

Using these data sets, we determined reference intervals for serum calcium in the patients selected for 0- to 90-day-old infants [7.8–11.3 mg/dL (1.9–2.8 mmol/L)]; 91- to 180-day-old infants [8.8–11.2 mg/dL (2.2–2.8 mmol/L)], 181- to 365-day-old children [8.8–11.4 mg/dL (2.2–2.9 mmol/L)], 1- to 3-year-old children [8.8–11.1 mg/dL (2.2–2.8 mmol/L)], 4- to 11-year-old children [8.8–10.7 mg/dL (2.2–2.68 mmol/L)], 12- to 19-year-old children [8.5–10.6 mg/dL (2.1–2.7 mmol/L)], patients younger than 19 years of age [8.6–10.9 mg/dL (2.2–2.7 mmol/L)] and patients older than 19 years old [8.6–10.9 mg/dL (2.2–2.7 mmol/L)] (Table 1 and Figure 1). For comparison, we generated calcium reference intervals for unselected subjects: 0- to 90-day-old infants [7.1–11.1 mg/dL (1.8–2.8 mmol/L)]; 91- to 180-day-old infants [7.6–11.0 mg/dL (1.9–2.8 mmol/L)], 181- to 365-day-old children [7.3–10.9 mg/dL (1.8–2.7 mmol/L)], 1- to 3-year-old children [7.6–10.5 mg/dL (1.9–2.6 mmol/L)], 4- to 11-year-old children [7.5–10.4 mg/dL (1.9–2.6 mmol/L)], 12- to 19-year-old children [7.3–10.2 mg/dL (1.8–2.6 mmol/L)], and patients older than 19 years old [7.2–10.2 mg/dL (1.8–2.6 mmol/L)]. A two-way ANOVA with Tukey's correction showed significant differences between the lower limits of normal (*P* < .001) and the normal range (*P* < .001), but not for the upper limit of normal, between these two groups.

**Table 1.** Age-Specific Reference Intervals of Serum Calcium Concentrations in Milligrams Per Deciliter

Age Range	Values Eliminating Patients on the Renal Unit or Endocrine Unit as Well as ICU (NICU, PICU and CICU) Patients			Values for Subjects From All Units With All 25(OH)D	Values for All Subjects (With Or Without 25(OH)D Concentrations)
	25(OH)D >30 and <80 (75–200 nmol/L )	25(OH)D Between 20 and 80 (50–200 nmol/L)	25(OH)D Between 20 and 30 (50–75 nmol/L)		
Birth to 90 d	8.0–11.3, n = 78 (2.0–2.8 mmol/L)	7.8–11.3, n = 110 (2.0–2.8 mmol/L)	7.5–11.1, n = 32 (1.9–2.8 mmol/L)	7.5–11.6, n = 140 (1.9–2.9 mmol/L)	7.1–11.1, n = 14,659 (1.8–2.8 mmol/L)
91–180 d	8.9–11.2, n = 96 (2.2–2.8 mmol/L)	8.8–11.2, n = 124 (2.2–2.8 mmol/L)	8.6–10.9, n = 28 (2.2–2.7 mmol/L)	8.5–11.2, n = 164 (2.1–2.8 mmol/L)	7.6–11.0, n = 5657 (1.9–2.8 mmol/L)
181–364 d	9.0–11.3, n = 163 (2.3–2.8 mmol/L)	8.8–11.4, n = 192 (2.2–2.9 mmol/L)	8.1–11.3, n = 29 (2.0–2.8 mmol/L)	8.2–11.6, n = 231 (2.1–2.9 mmol/L)	7.3–10.9, n = 5952 (1.8–2.7 mmol/L)
1–3 y	8.9–11.1, n = 568 (2.2–2.8 mmol/L)	8.8–11.1, n = 756 (2.2–2.8 mmol/L)	8.5–11.0, n = 188 (2.1–2.8 mmol/L)	8.6–10.8, n = 832 (2.2–2.7 mmol/L)	7.6–10.5, n = 16708 (1.9–2.6 mmol/L)
4–11 y	8.7–10.7, n = 962 (2.2–2.7 mmol/L)	8.8–10.7, n = 1440 (2.2–2.7 mmol/L)	8.6–10.7, n = 478 (2.2–2.7 mmol/L)	8.4–10.6, n = 2186 (2.1–2.7 mmol/L)	7.5–10.4, n = 27102 (1.9–2.6 mmol/L)
12–19 y	8.5–10.7, n = 943 (2.1–2.7 mmol/L)	8.5–10.6, n = 1716 (2.1–2.7 mmol/L)	8.5–10.5, n = 773 (2.1–2.6 mmol/L)	8.2–10.5, n = 2788 (2.1–2.6 mmol/L)	7.3–10.2, n = 30909 (1.8–2.6 mmol/L)
>19 y	8.5–10.5, n = 195 (2.2–2.6 mmol/L)	8.5–10.5, n = 27 (2.1–2.6 mmol/L)	8.4–10.4, n = 92 (2.1–2.6 mmol/L)	8.2–10.3, n = 526 (2.1–2.6 mmol/L)	7.2–10.2, n = 5223 (1.8–2.6 mmol/L)
Birth to 19 y	8.6–10.9, n = 2810 (2.2–2.7 mmol/L)	8.6–10.9, n = 4338 (2.2–2.7 mmol/L)	8.5–10.7, n = 1528 (2.1–2.7 mmol/L)	8.3–10.7, n = 6341 (2.1–2.7 mmol/L)	
All ages	8.6–10.9, n = 3008 (2.2–2.7 mmol/L)	8.8–10.8, n = 4629 (2.2–2.7 mmol/L)	8.5–10.7, n = 1621 (2.1–2.7 mmol/L)	8.2–10.7, n = 6867 (2.1–2.7 mmol/L)	7.4–10.6, n = 106220 (1.9–2.7 mmol/L)

Abbreviation: NICU, neonatal intensive care unit; PICU, pediatric intensive care unit. Values include interval, n, below in parentheses (in nanomoles per liter).

Within the group of selected patients, we attempted to determine a physiological lower limit of normal for serum 25(OH)D based on observation of an inflection point in serum calcium concentration. To do this, we attempted to fit a dose-response curve to all calcium values by 25(OH)D concentrations. This curve revealed an inflection point at 28.4 ng/mL (70.9 nmol/L) with 95% confidence intervals ranging from 27.7 to 29.0 (69.1–72.3 nmol/L) and an R^2^ value of 0.022 (Figure 1). Additionally, based on the controversy of whether 20 or 30 ng/mL (50 or 75 nmol/L) is the appropriate lower limit of normal for 25(OH)D, we generated new reference intervals based on two groups: those with a 25(OH)D from 20 to 30 ng/dL (50–75 nmol/L) comprised one group, and those with a 25(OH)D from 30 to 80 ng/dL (75–200 nmol/L) comprised the second group. Within this cohort, serum 25(OH)D levels were between 20 and 30 ng/mL (50–75 nmol/L) in 31% of subjects; *t* tests revealed significant differences at all pediatric ages aside from greater than 19 between calcium concentrations in these two groups (Figure 1B). Furthermore, paired *t* tests showed a significant [two tailed *P* < .03, difference 0.24 mg/dL (0.06 mmol/L)] between the lower limits of normal for these subjects as well as the upper limits [two tailed *P* < .002, difference 0.12 mg/dL (0.03 mmol/L)] but no difference in range compared with subjects with 25(OH)D levels between 30 and 80 ng/mL (75 and 200 nmol/L). These results support the proposition, shown in prior studies based on serum PTH levels (8) and calcium absorption efficiency (9, 10), that 30 ng/mL (75 nmol/L) is a physiological lower limit of normal for serum 25(OH)D.

## Discussion

Here we report age-adjusted reference intervals for total serum calcium concentration based on apparently normal subjects with vitamin D sufficiency. Similar to previous studies, we found that serum calcium levels were greatest in younger subjects, and that the upper limits of the reference ranges declined with age (11, 12). We found that at all ages, the lower limit of the reference intervals for normal subjects with vitamin D sufficiency [ie, 25(OH)D levels greater than 20 ng/ml (50 nmol/L)] were significantly greater (*P* < .001) than those for unselected subjects. By contrast, the upper limit of these reference ranges did not vary between groups defined by vitamin D status. Hence, the reference intervals for serum calcium that we have determined likely reflect a more physiologically appropriate reference interval, rather than a reference range based on values from an unselected population that includes many subjects with vitamin D insufficiency. That is, the increase in precision in the normal range of serum calcium actually reflects an increase in accuracy. Our observations suggest that many subjects with 25(OH)D levels less than 30 ng/mL have mild hypocalcemia. We did not observe a relationship between 25(OH)D and higher levels of total serum calcium, consistent with the notion that within the physiological range, levels of 25(OH)D do not lead to higher concentrations of serum calcium in patients with intact parathyroid gland function. Most importantly, our results indicate that inclusion of patients with low serum levels of 25(OH)D has a significant impact on the determination of reference ranges for serum calcium.

Our studies also addressed the ongoing controversy in the field of vitamin D biology concerning the definition of vitamin D deficiency. Several attempts to define the lower limit of the normal range for 25(OH)D have relied on population data showing an increase in serum levels of PTH when serum levels of 25(OH)D are less than 30 ng/mL (75 nmol/L) (13, 14). Mortality data, as well as the difficulty in determining the inflection point of the PTH curve, have limited acceptance of these results, however (15–17). Based on other studies, some authorities (15, 18) have proposed that a serum concentration of 25(OH)D less than 20 ng/dL represents a state of vitamin D deficiency. Our data indicate that many patients with serum 25(OH)D concentrations between 20 and 30 ng/mL have mildly depressed serum calcium levels and thereby support the premise that 30 ng/mL represents a more valid lower physiological limit.

There are several weaknesses to this study. We cannot be certain that our patients are really apparently healthy because many hospitalized children were acutely ill, which may have influenced serum calcium or 25(OH)D levels. Moreover, we did not use albumin levels to adjust calcium, nor we did not measure serum levels of vitamin D binding protein or PTH. Although we did not adjust 25(OH)D concentrations for season, we included measurements obtained throughout the year. Finally, because of the retrospective nature of our study, we had no knowledge of calcium or vitamin D intake for any of the patients. However, the strengths of this study include the large number of patients and an extensive data set, a broad representation of age spectrum, and inclusion of a diverse and free-living patient population comprised of all ethnic groups.

Our reference intervals are considerably broader than other recently published pediatric calcium reference intervals (11, 12). This broadened dispersion, in the context of a significantly greater number of values, may reflect seasonal heterogeneity or ethnic heterogeneity because the previously reported values were reported for ethnically homogenous groups (11, 12). Nevertheless, our study represents the first attempt to develop calcium reference ranges for a pediatric population that excludes subjects who are vitamin D deficient. These new reference intervals refine previous normal ranges that likely included many subjects who had abnormal vitamin D status and suggest that future studies to define the reference intervals for normal serum calcium levels should consider vitamin D status in the selected cohort.
